# Effect of brewer’s yeast or beta-glucan on breast milk supply following preterm birth: the BLOOM study – protocol for a multicentre randomised controlled trial

**DOI:** 10.1186/s13006-024-00650-z

**Published:** 2024-06-20

**Authors:** Luke E. Grzeskowiak, Alice R. Rumbold, Lauren Williams, Renee L. Kam, Wendy V. Ingman, Amy Keir, Kathryn A. Martinello, Lisa H. Amir

**Affiliations:** 1 College of Medicine and Public Health, Flinders Health and Medical Research Institute, Flinders University, Flinders Drive, Bedford Park, SA 5042 Australia; 2 Flinders Medical Centre, SA Pharmacy, SA Health, Bedford Park, SA Australia; 3 Women and Kids Theme, South Australian Health and Medical Research Institute, North Adelaide, SA Australia; 4 Adelaide Medical School, University of Adelaide, Adelaide, SA Australia; 5 Judith Lumley Centre, School of Nursing & Midwifery, La Trobe University, Bundoora, VIC Australia; 6 Discipline of Surgical Specialities, Adelaide Medical School, University of Adelaide, The Queen Elizabeth Hospital, Woodville, Australia; 7 Robinson Research Institute, University of Adelaide, Adelaide, Australia; 8 Department of Neonatal Medicine, Women’s and Children’s Hospital, North Adelaide, SA Australia; 9 Department of Neonatal and Perinatal Medicine, Flinders Medical Centre, Bedford Park, SA Australia; 10 Breastfeeding Service, The Royal Women’s Hospital, Parkville, VIC Australia

**Keywords:** Lactation, Breastfeeding, Breast milk expression, Breast milk production, Breast milk supply preterm birth, Galactagogue, Brewer’s yeast

## Abstract

**Background:**

Many individuals who experience preterm birth struggle with early breast milk supply, which can translate into suboptimal longer-term breastfeeding outcomes. Further investigations into the potential role of early non-pharmacological and pharmacological interventions in improving breast milk production soon after birth is growing. While natural galactagogues, such as brewer’s yeast, are widely perceived by women to be safer than pharmaceutical galactagogues and are taken by many women, evidence to support their efficacy is largely absent. The BLOOM study has been designed to determine the efficacy and safety of brewer’s yeast and beta-glucans, derived from *Saccharomyces cerevisiae*, when administered soon after birth for increasing early breast milk supply in mothers who have delivered preterm.

**Methods:**

The BLOOM study is a multicentre, double-blinded, randomised controlled trial that will assess if brewer’s yeast or beta-glucan can increase early breast milk production following preterm birth. Target population are mothers of preterm infants born at less than 34 weeks’ gestation who intend to provide breast milk for their infant, are less than 72 h following birth and able to give informed consent. Participants will be randomly allocated into three parallel groups at 1:1:1 ratio (*n* = 33 per group) to receive either brewer’s yeast, beta-glucan or placebo capsules for seven days. The primary outcome is total expressed breast milk volume over a 24-hour period on day 7 of intervention. Participants and their infants will be followed until the infant reaches term corrected age or is discharged home from the neonatal unit (whichever occurs first).

**Discussion:**

The use of brewer’s yeast as a galactagogue to enhance milk production is extremely common amongst breastfeeding mothers, however, there are no trials evaluating its efficacy and safety. This will be the first randomised controlled trial to evaluate the efficacy and safety of two commonly used galactagogues, brewer’s yeast and beta-glucan, compared with placebo in improving maternal breast milk supply following preterm birth. The trial will also evaluate whether early intervention with galactagogues soon after a preterm birth improves longer-term breastfeeding outcomes.

**Trial registration:**

Australian and New Zealand Clinical Trials Registry ACTRN12622000968774 (registered on 8 July 2022) and UTN U1111-1278-8827.

## Background

Breast milk is considered the optimal form of enteral nutrition for preterm infants [1, 2]. There is a compelling body of evidence demonstrating that the provision of mothers’ own breast milk significantly reduces neonatal morbidity and mortality [3, 4]. Specifically, use of mothers’ own breast milk during hospitalisation reduces the incidence and severity of certain morbidities, including necrotizing enterocolitis (NEC), late onset sepsis, chronic lung disease, retinopathy of prematurity, rehospitalisation after discharge, and neurodevelopmental problems in infancy and childhood [3]. A recent Cochrane review showed preterm infants receiving infant formula are three times more likely to develop NEC, which has a mortality rate of 20–40% [5]. Further, the ability for a mother to provide her own breast milk represents an important tangible contribution in a situation where mothers are separated from their infant during the neonatal hospitalisation and may have limited opportunities to directly participate in infant care in the first few weeks of life [6–8]. The resultant psychological benefits can include greater feelings of attachment, empowerment, and confidence [9].

Despite the noted benefits, mothers of preterm infants face many challenges in establishing and maintaining an adequate supply of breast milk during their infant’s prolonged hospitalisation. This is driven by multiple factors including physiological immaturity of the breast associated with preterm birth, inability for the preterm infant to breastfeed directly from the breast, and the stress of having an infant admitted to a Neonatal Intensive Care Unit (NICU) [10]. Each of these factors has the ability to interfere with establishment of normal milk supply; one previous study demonstrated 82% of women birthing preterm experienced delayed secretory activation [11]. While longer-term breastfeeding outcomes were not collected for this cohort, other studies in mothers of term infants have demonstrated that delayed secretory activation is associated with increased risk of early cessation of breastfeeding [12].

Some mothers may respond well to non-pharmacological lactation support strategies (i.e. correct use of breast milk pump, increasing expressing frequency) for increasing breast milk supply, but for a large number of preterm mothers, their breast milk supply continues to be insufficient to meet their infant’s needs. Insufficient volume of mothers’ own breast milk has been partly addressed through the introduction of human milk banks, however donor milk is not universally available. Further, mothers’ own milk has been demonstrated to be superior to donor human milk with respect to composition and bioactivity, highlighting that focusing on supporting mothers to provide their own breast milk to their infants is key to optimising neonatal outcomes [3].

Given the challenges mothers of preterm infants face in initiating and sustaining lactation, attention has shifted towards the potential role of early non- pharmacological and pharmacological interventions in improving breast milk production soon after birth. A recent survey of 1876 Australian women found that 60% reported taking galactagogues, known as substances thought to aid in initiating and maintaining adequate milk production, during breastfeeding [13]. In 2023, galactagogues were also reported as being used in over half of the participants in an online survey of breastfeeding mothers in the United States [14]. This is despite a lack of clear evidence to guide their use [15, 16]. The most commonly reported galactagogues in recent surveys include lactation cookies, oats, and brewer’s yeast [13]. While lactation cookies may vary in composition, they typically consist of ingredients such as oats, brewer’s yeast and flaxseed. The active ingredient of oats or brewer’s yeast is thought to be beta-glucan, which is a glucose polymer present in the cell walls of cereals as well as yeast (i.e. brewer’s yeast) and fungi [17].

While natural galactagogues, such as brewer’s yeast, are widely perceived by women to be safer than pharmaceutical galactagogues and are taken by many women [13], evidence to support their efficacy in increasing breast milk production is largely absent. Furthermore, natural galactagogues have been used to treat lactation insufficiency but appear to be increasingly used prophylactically. Evidence to support their use either in the treatment or prevention of low milk supply following preterm birth is lacking. In the survey conducted by Ryan et al., Brewer’s yeast was the galactagogue participants felt had the greatest impact on increasing breast milk supply [14]. With respect to brewer’s yeast derived from *Saccharomyces cerevisiae*, the likely mechanism of action remains unknown. Some have postulated that it could relate to the high concentration of B vitamins, or the presence of beta-glucan isolated from the cell wall [18]. Further, a number of studies have demonstrated that oral consumption of beta-glucans may have immunomodulatory effects [17]. As immune dysregulation is a common feature of preterm birth [19] and has been associated with impaired lactation in animal models [20], this represents an alternative potential mechanism of action in which brewer’s yeast/ beta-glucans could influence breast milk production. Data in lactating women, however, remains scant.

A recent clinical trial by Wesolowska et al., evaluated the efficacy of a barley malt-based galactagogue (containing a proprietary blend of barley malt and beta-glucan) compared with placebo in mothers of preterm infants [21]. Compared with placebo, those who took the barley malt preparation reported expressing a greater total volume of breastmilk over the 14-day intervention period (6036 ± 498 vs. 4209 ± 335; *p* = 0.003). Differences in daily expressed breast milk volume between groups were evident by day seven of the intervention. No participants reported experiencing any side effects during the study. However, the study can only be considered to provide low quality evidence due to a substantial loss to follow-up of 32% across both treatment groups. The beta-glucans found in cereals are also noted to be structurally different and exert different physiological effects to those founds in yeast, therefore the generalisability of these findings to brewer’s yeast are uncertain. Given the scant literature, it remains unknown whether natural galactagogues such as brewer’s yeast or beta glucan are effective in improving breast milk production following preterm birth.

The BLOOM Study (Can *Brewer’s* yeast or beta-g*L*ucan increase m*O*ther’s *O*wn *M*ilk supply following preterm birth?) will evaluate if routine administration, commenced within the first week postpartum, of brewer’s yeast or beta-glucan, both derived from *Saccharomyces cerevisiae*, increases early breast milk production following preterm birth.

### Objectives

This main objective of this study is to determine whether in mothers of a preterm infant born at less than 34 weeks’ gestation, does taking brewer’s yeast or beta-glucan compared with placebo lead to an increase daily expressed breast milk volume after seven days.

## Methods

### Study design and setting

The BLOOM study is a randomised, controlled, clinician, researcher and participant/family blinded, multicentre trial with three parallel groups with a 1:1:1 allocation ratio. The sponsoring institution and trial coordinating centre is the South Australian Health and Medical Research Institute (SAHMRI) based at the Women’s and Children’s Hospital. The study will take place in three locations within Australia (Women’s and Children’s Hospital and Flinders Medical Centre, South Australia and The Royal Women’s Hospital, Victoria). Participant screening, enrolment, randomisation, and study visits will be undertaken locally at each study site. A trial steering committee consisting of LEG, LHA and LW will oversee all clinical trial activities. The study protocol was prepared following the SPIRIT guideline and checklist [22].

### Study population

Mothers of preterm infants born at less than 34 weeks’ gestation who intend to provide breast milk for their infant, and are within 72 h of giving birth.

The inclusion criteria will include women, 18 years or older, who have delivered a preterm infant born at less than 34 weeks’ gestation (up to 33 + 6), are between 0 and 72 h of birth, intend to provide breast milk to their preterm infant for any desired duration, and able to give informed consent. Women with a contraindication to breastfeeding such as infection with human immunodeficiency virus (HIV), as well as those with higher order pregnancies (triplet or more) will be excluded from the study.

**Fig. 1:**
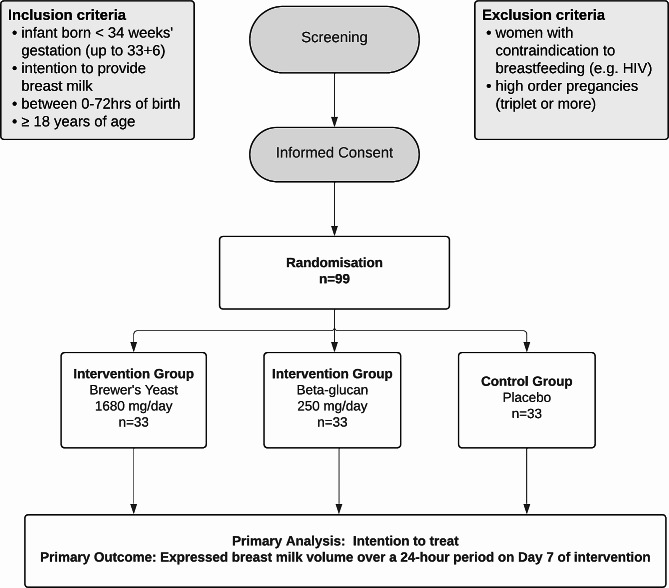
BLOOM study participant flow throughout the study

Information on the study will be provided to eligible participants by their nurse or midwife who will ask them to complete a Consent to Contact form, and following this consent will be contacted by a study researcher (Fig. 1). After confirmation of eligibility and receipt of voluntary informed consent, women will be assigned randomly to one of three groups: brewer’s yeast, beta-glucan, or placebo in a 1:1:1 ratio using randomly permuted blocks of varying sizes. Block sizes will not be disclosed to ensure allocation concealment. Participants will be randomised using the REDCap Randomisation Module, and the randomisation schedule will be prepared using ralloc. ado in Stata by an independent statistician that is not involved with trial participants or data. The randomisation schedule will be stratified according to study site. Participants and their care providers, outcome assessors, trial investigators and data analysts will be blinded to randomisation group. Women will be allocated a random, unique, re-identifiable randomisation identification number (ID) and assigned to one of three treatment groups and be issued with study medications identified by colour. To facilitate blinding, two colours will be used for each treatment group, with six colours used in total (i.e. green, yellow, red, blue, purple, orange).

Women will be free to withdraw themselves and/or their infant from the study at any time.

Participant recruitment for the BLOOM trial began in July 2022 and recruitment is expected to be completed in mid-2024.

### Intervention

Participants will be randomised to one of three treatment arms, consisting of either brewer’s yeast (*Saccharomyces cerevisiae*), beta-glucan (purified from *Saccharomyces cerevisiae*) or placebo (microcrystalline cellulose), study medications will be manufactured and supplied by Leiber GmbH (Germany). To maintain study blinding, all study participants will take an oral dose of three capsules twice daily (six capsules a day) according to the treatment schedule indicated in Table 1 for a total of seven days from enrolment. To facilitate treatment blinding and aid medication adherence, the medications will be provided in bottles with instructions according to the dosing regimen (Table 1) and all study medications will be identical in appearance. Study treatment will be discontinued if either of the following occur: unacceptable toxicity; treatment continuation is determined not to be in the participants’ best interest; or participant declines taking study medication or withdraws their consent.

**Table 1:** BLOOM study treatment dosing regimen

Treatment Arm	Morning	Evening	Total amount active ingredient per day
Brewer’s Yeast	3 × 280 mg capsules	3 × 280 mg capsules	1680 mg
Beta-glucan	1 × 250 mg capsule 2 x placebo capsules	3 x placebo capsules	250 mg
Placebo (Microcrystalline cellulose)	3 x placebo capsules	3 x placebo capsules	-

Medication adherence will be monitored, with participants asked to document all doses taken in a provided breast milk diary. Participants will be able to access and receive lactation support as necessary and as per local hospital policies during the trial. Given the absence of any known drug-drug interactions, no restrictions will be placed on the concomitant use of other medications including use of other galactagogues.

### Patient and public involvement

We received input from the SAHMRI Women and Kids consumer group, comprising women with young children, to ensure members of the public were involved at several stages of the trial, including design, management, and conduct. The burden of the trial was carefully assessed. We intend to disseminate the main results to trial participants and will seek patient and public involvement in the development of an appropriate method of dissemination.

### Outcomes

#### Primary outcome

**Fig. 2:**
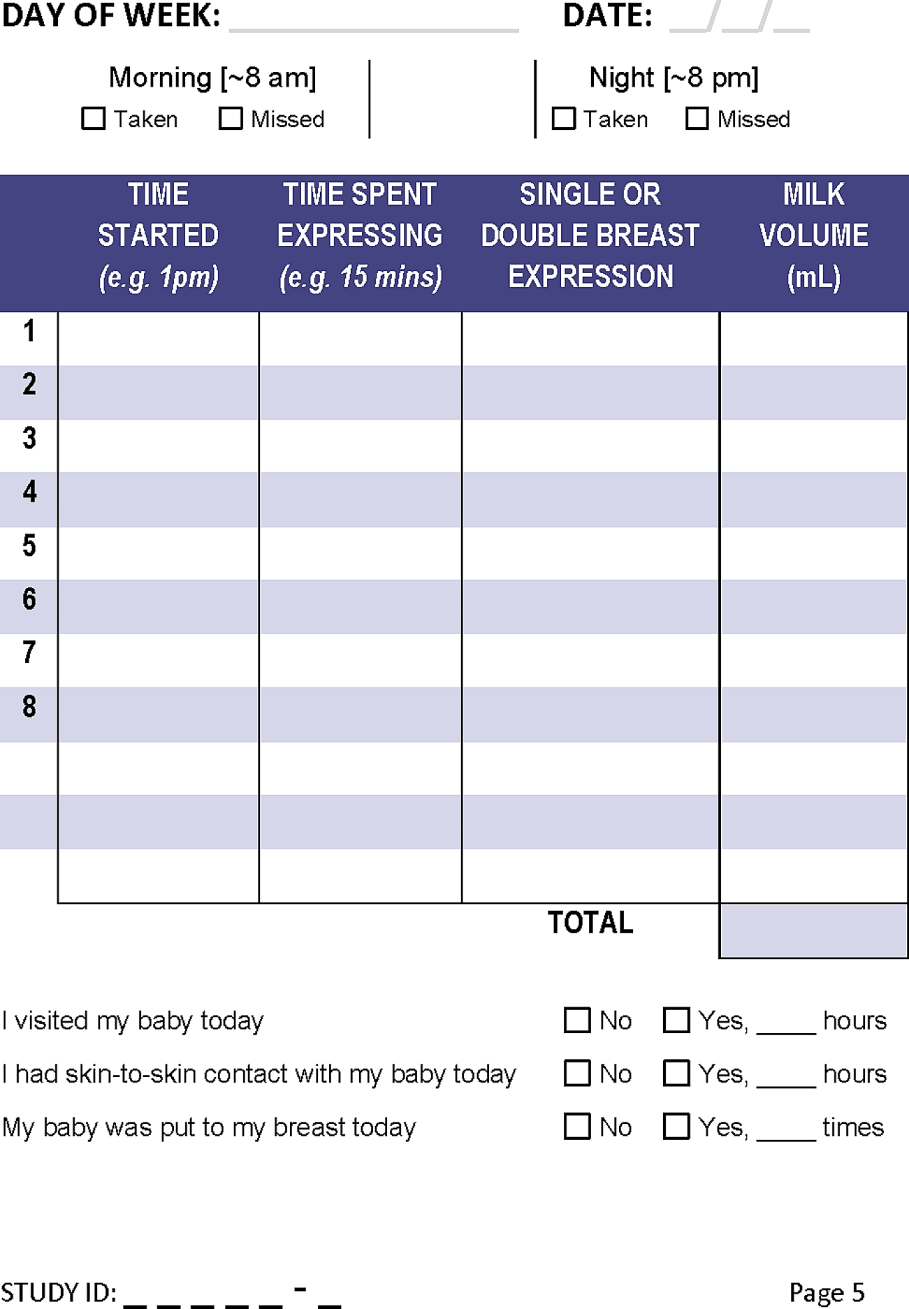
Excerpt of BLOOM study breast milk diary

Daily expressed breast milk volume on Day 7 post study intervention. Following randomisation all participants will be asked to maintain a daily breast milk diary throughout each day of the study (Fig. 2). The diary will act as a record (self-reported) of each time breast milk was expressed, time spent expressing, whether they expressed one breast at a time or both breasts simultaneously, and the volume of expressed milk. Each individual volume of expressed milk will be summed to generate a total for expressed breast milk over a 24-hour period (midnight to midnight).

#### Secondary outcomes

##### Maternal breastfeeding outcomes

Infant intake of supplemental enteral feeds (donor milk or infant formula), proportion of enteral feeds consisting of mother’s own breast milk, total duration and extent of breast milk expression and/or direct breastfeeding up until infant discharge or term corrected age (whichever occurs first), total expressed breast milk volume during study intervention, total expressed breast milk volume up to and on day 21 postnatal. Data on breast health will be assessed using a modified breast health questionnaire, while data on breast milk expression will be assessed using the Breast Milk Expression Experience (BMEE) questionnaire [23]. We will also collect data on self-reported use of other galactagogues.

##### Maternal outcomes

Maternal mental health and wellbeing will be assessed using three validated instruments: Edinburgh Postnatal Depression Scale (EPDS) [24], Six-item State-Trait Anxiety Inventory (STAI-6) [25], and the Parental Stressor Scale: Neonatal Intensive Care Unit (PSS: NICU) [26]. Impacts of the intervention on maternal and infant bonding will be assessed using the Maternal Postnatal Attachment Scale (MPAS) [27].

##### Infant outcomes

Differences in infant anthropometrics (weight, length, head circumference) at discharge from hospital or term corrected age (whichever occurs earlier), trajectories of infant anthropometrics from birth (weight, length, head circumference) to discharge from hospital or term corrected age (whichever occurs earlier). Data on infant morbidity and mortality will be compared based on information obtained from the Australian and New Zealand Neonatal Network (ANZNN) registry.

##### Safety outcomes

Safety outcomes including side effects and tolerability of study medications will be assessed through routine data collection by maternal self-report. Serious adverse events will be assessed: maternal mortality; maternal hospitalisation; or other life-threatening events.

##### Biological outcomes

A range of biospecimens will be collected as part of the study as outlined in Tables 2 and 3. Samples will be stored for future analysis relating to this study which may include investigating changes in breast milk macronutrient composition; differences in the faecal microbiome; differences in cytokines and other proteins in breast milk and/or blood; differences in serum prolactin and markers of secretory activation (i.e. Na and K); differences in urinary metabolome.

**Table 2:** Overview of the BLOOM Study schedule of assessments

	Day 0 Baseline	Day 7 Post enrolment	Day 21 Postpartum	Infant Discharge to Home or Term Corrected Age
**Questionnaires**				
General				
- Maternal demographic & lifestyle questionnaire	X			
- Postnatal health		X	X	X
Breastfeeding				
- Daily breast milk diary	X	X	X	
- General breast milk expression and breastfeeding questions	X	X	X	X
- Breast health questionnaire		X	X	X
- Breast Milk Expression Experience questionnaire		X		X
Mental health and wellbeing				
- EPDS		X		X
- STAI-6		X		X
- PSS: NICU		X		X
- MPAS				X
Medication side-effects		X	X	
**Physical assessments**				
Maternal anthropometry		X		
Maternal biospecimens*				
Blood		X		
Urine		X		
Stool		X		
Buccal swab		X		
Breast milk		X		
**Case note audit**				
Pregnancy and birth history	X			
Infant feeding record	X	X	X	X
Infant anthropometry	X	X	X	X
Infant morbidity and mortality				X
* Protocol window*	-	*Day 7–9*	*Day 21–23*	*Within 3 days of event*

EPDS: Edinburgh postnatal depression scale, STAI-6: Six-item State-Trait Anxiety Inventory, PSS:NICU: Parental Stressor Scale: Neonatal Intensive Care Unit, MPAS: Maternal Postnatal Attachment Scale

*All biological samples are collected for research purposes only

**Table 3:** Biospecimen sample collection at visit 2 (Day 7 following intervention)

Sample type	Description
Maternal blood	• 30 mL blood (Serum tube 10 mL, K2 EDTA 10 mL and 5 mL, and Trace Elements 5 mL) • Cryopreservation of plasma trace element, serum, plasma, buffy coat, red blood cells aliquots • Collected between 10 am and 12pm
Breast milk	• Expressed milk (3–8 mL) from a single breast expression episode • Cryopreserved aliquots of whole milk
Maternal urine	• 25 mL urine • Cryopreserved aliquots
Maternal stool	• 1x Collection pot (Norgen Biotek) • Cryopreserved sample pot
Maternal buccal swab	• FloqSwab (Copan Diagnostics) • Air-dried swab cryopreserved • Researcher collected

### Data and sample collection

Data will be collected from all participants across four study time-points (Day 0 – Baseline, Day 7 post intervention, Day 21 postpartum, and at infant discharge or at term corrected age) as outlined in Table 2.

Questionnaires and assessments will be completed at study visits as described in Table 4 at the corresponding timepoints indicated in Table 2. Biological samples will be collected for research purposes only as indicated in Tables 2 and 3. Appointments will be conducted within the hospital (either within the neonatal unit, postnatal ward, or designated clinical research area), or by telephone (when required, e.g. if mother is not visiting on the day or illness prevents a face-to-face visit).

**Table 4:** Overview of data collected as part of the BLOOM study

Questionnaire or assessment	Description
**Questionnaires/Assessment**	
General information	Contact information, including name, address, phone number, hospital ID number.
Maternal demographic and lifestyle information	Maternal age, height, pre-pregnancy weight, smoking status, alcohol intake, pre-existing medical conditions, ethnicity, country of birth, income status, education level, marital status, previous pregnancy and breastfeeding history, pregnancy complications, medication use, postnatal contraception use, breast type/shape, breast development since puberty and during pregnancy.
Breast milk diary	Daily breast milk diary recording the number of times each breast was expressed, the method used to express, the volume of expressed milk, duration and if applicable information pertaining to direct breastfeeding for a total of 21 days.
Breast milk expression	Intended duration of breastfeeding, breast development previous to and during pregnancy, time of first expressing following birth, perceived onset of lactogenesis, methods and type of breast milk expressing (including type of breast milk pump), duration of breast milk expressing or breastfeeding, and provision of lactation support.
Breast Health Questionnaire	Questionnaire adapted from Fetherston 2006 to assess general breast health and potential risk for mastitis. Collects information on breast fullness, breast pain and discomfort, duration of pain/discomfort, presence of cracks or grazes on the nipples, fever, as well as any areas of heat, swelling or redness on breasts.
Breast Milk Expression Experience (BMEE) Questionnaire	BMEE provides a measure of milk expression experience across three dimensions: (1) social support for milk expression; (2) ease of learning how to express milk; and (3) personal experiences of milk expression.
Postnatal Health Questionnaire	Postnatal health issues during the study (e.g. infections, cold/flu), as well as any medications (prescription or non-prescription), herbal supplements or multivitamins taken.
Mental Health and Wellbeing Questionnaires • EPDS • STAI-6 • PSS: NICU • MPAS	Assessment of depression and anxiety symptoms will be collected using standardises validated questionnaires (Edinburgh Postnatal Depression Scale [EPDS], Spielberger State-Trait Anxiety Inventory [STAI-6]). Stress related to infant hospitalisation will be assessed using the Parental Stressor Scale: NICU (PSS: NICU). Maternal-infant attachment will be assessed using the Maternal Postnatal Attachment Scale (MPAS).
Maternal anthropometry	Maternal weight, height, waist circumference, hip circumference, and mid upper-arm circumference will be assessed.
Medication side-effects and adverse event	Recording any side-effects pertaining to potential adverse events or serious adverse events. Details encouraged to be recorded in breast milk diary too.
**Case Note Data Extraction**	
Pregnancy and birth history	Gravida, parity, length of gestation, infant birthweight, infant sex, plurality, onset of labour, method of birth, estimated blood loss, pregnancy complications (i.e. gestational diabetes, gestational hypertension, pre-eclampsia), antenatal interventions (i.e. receipt of antenatal corticosteroids of magnesium sulphate), medication use, antenatal haematology (i.e. haemoglobin, iron studies).
Infant feeding record	Daily record of total enteral intake, volume of expressed breast milk, donor milk, or infant formula, as well as proportion of enteral feeds consisting of mothers’ own breast milk.
Infant anthropometry	Weight, length, head circumference.
Infant morbidity/mortality	Infant morbidity and mortality data will be obtained from the Australian and New Zealand Neonatal Network (ANZNN) [29] registry. Data include, number of days in hospital (to first discharge home), intraventricular haemorrhage (IVH), confirmed sepsis, necrotizing enterocolitis (NEC), retinopathy of prematurity (ROP), surgical procedures and death during initial hospitalisation.

### Sample size calculation

A sample of 99 women (33 per arm) yields 90% power, 0.025 alpha to show a difference in the mean daily breast milk volume of 150 mL/day (200 mL/day standard deviation) between each of the intervention arms and control arm, allowing for 10% loss to follow-up. This includes adjustment for a 0.6 correlation between breast milk volume at study entry and breast milk volume on Day 7 at the end of the intervention phase.

### Data management

Data entry and study management will be using a purpose-built REDCap database and hosted on secure servers at SAHMRI. Electronic case report forms will be used and stored directly in REDCap, and any paper-based case report forms will be stored in a locked office at the study site. Data are collected by trained researchers and entered directly into REDCap, no confidential data are stored on data entry machines. REDCap uses a MySQL database via a secure web interface and includes a complete suite of features to support the Health Insurance Portability and Accountability Act of 1996 compliance, including a full audit trail, user-based privileges and integration with the institutional Lightweight Directory Access Protocol server [28].

In order to adhere to international best research practice and to ensure eligibility of our study for publication in scientific journals, we plan to archive the fully deidentified sequencing datafiles in the Gene Expression Omnibus (GEO or equivalent database). The sequencing data files prepared for GEO will be free of all identifiers that would permit linkages to individual research participants and variables that could lead to deductive disclosure of the identity of individual subjects, in accordance with the NIH Statement on Sharing Research Data (2003).

### Statistical analysis methods

The primary analysis will be performed according to the treatment group to which participants were randomised (intention-to-treat principle). A secondary per-protocol analysis will also be performed for each of the primary and secondary outcomes.

The primary outcome of daily expressed breast milk volume on Day 7 post intervention will be compared between treatment groups using linear regression. The results will be expressed as a difference in means with a 95% confidence interval and two-sided *p*-value. Adjustment will be made for baseline daily expressed breast milk volume and the randomisation strata (study centre). A *p*-value of less than 0.05 will be considered to indicate statistical significance. Analysis of secondary outcomes will use log-binomial regression models for binary outcomes and linear regression models for continuous outcomes with adjustment for stratification variables and other pre- specified prognostic baseline variables. Results will be presented as relative risks and differences in means respectively, along with 95% confidence intervals. Missing data will be addressed using multiple imputation. Sensitivity analyses will also be performed using the original unimputed data.

Planned sub-group analyses of the primary and secondary breastfeeding outcomes include: (i) Plurality (singleton vs. twins); (ii) Parity (primiparous vs. multiparous); (iii) Infant gestation at birth (categorical: 23^+ 0^–29^+ 6^ vs. 30^+ 0^–31^+ 6^ vs. 32^+ 0^-33^+ 6^ weeks’ gestation and continuous); (iv) Maternal body mass index (categorical: underweight/normal weight [BMI < 25 kg/m^2^] vs. overweight/obese [BMI ≥ 25 kg/m^2^] and continuous). Effect modification by each of these factors will be assessed separately by including interaction effects with treatment group in the statistical models.

### Data monitoring and safety

The trial coordinating centre (SAHMRI) will perform ongoing monitoring via weekly reports of participants screened/enrolled/randomised, visits due/overdue/missed, adverse and serious adverse events, product inventory and dispense records, sample collection logs, and participant communication logs. The weekly reports will be reviewed at weekly trial management meetings. No interim analyses are planned.

An individual participant may only be unblinded in emergency situations, where the Investigator decides a participant cannot be adequately treated without knowing the identity of their treatment allocation. To break the randomisation code the Principal Investigator must contact the randomisation facility/personnel. The time, date, participant study ID and reason for unblinding will be documented. Events leading to the emergency breaking will be recorded in the serious adverse event (SAE) report form.

### Ethical issues and dissemination

The BLOOM study will be conducted in accordance with the approved version of the Study Protocol and in compliance with the Australian National Statement on Ethical Conduct in Research Involving Humans which builds on the ethical codes of the Declaration of Helsinki and the Principles of International Conference on Harmonisation Good Clinical Practice (as adopted in Australia). All study materials and study protocol (current version 1.1) have been reviewed and approved by the Women’s and Children’s Health Network Human Research Ethics Committee (HREC), South Australia (HREC/22/WCHN/00001), as well as the research governance officers at each of the study sites. Any change to the protocol or Informed Consent Form that affects the scientific intent, study design, patient safety or may affect a participant’s willingness to continue participation in the study is considered an amendment, and therefore, will be written and filed as an amendment to this protocol and/ or informed consent form. All such amendments will be submitted to the HREC, for approval prior to becoming effective.

The primary and key secondary breastfeeding objectives will be analysed and reported first. Study findings will be submitted for peer-reviewed publication and for presentation at appropriate local and international conferences. In addition, study findings will be disseminated to participants through a brief lay summary. No participants will be identified in the dissemination of study results and data collected will be treated with confidence.

### Access to data and study documentation

De-identified individual participant data will be made available upon reasonable request. Requests to access data must be reviewed and approved by both the BLOOM trial steering committee and the Women’s and Children’s HREC, and will be assessed for scientific and methodological rigour. Individuals requesting data will be required to sign a data access agreement. Requests for data or study documentation should be directed to the Chair of the BLOOM Steering Committee, LEG via email (luke.grzeskowiak@flinders.edu.au).

## Discussion

This study seeks to monitor the effectiveness and safety of consuming of galactagogues on breast milk production following preterm birth. Galactagogues in the form of brewer’s yeast or beta-glucan will be provided to participants and compared with a placebo group for a 7-day period. Recruitment commenced in July 2022 and will aim to be completed within two years.

### Strengths and limitations

The planned study has a number of strengths, notably the rigorous experimental design and comprehensive assessment across a range of maternal and infant outcomes up until the point of infant discharge home from hospital (or until term corrected age, whichever occurs first) However, the study also has some limitations. Firstly, estimation of total daily breast milk volume is based solely on expressed volumes. In the case that infants fed directly from the breast, pre- and post- feed weighing of the infant was not included due to the added burden and stress, and expected negligible overall impact on the primary study outcome given noted difficulties in establishing direct breastfeeding following preterm birth before 34 weeks’ gestation. Further, the use of infant test-weighing is not an established practice within any of the study trial sites.

## Data Availability

No datasets were generated or analysed during the current study.

## Abbreviations

ANZNN: Australian and New Zealand Neonatal Network
BLOOM: Can *Brewer’s* yeast or beta-g*L*ucan increase m*O*ther’s *O*wn *M*ilk supply following preterm birth?
BMEE: Breast Milk Expression Experience questionnaire
EPDS: Edinburgh Postnatal Depression Scale
HREC: Human Research Ethics Committee
MPAS: Maternal Postnatal Attachment Scale
NEC: Necrotizing enterocolitis
NICU: Neonatal Intensive Care Unit
PSS:NICU: Parental Stressor Scale: Neonatal Intensive Care Unit
SAHMRI: South Australian Health and Medical Research Institute
STAI-6: Six-item State-Trait Anxiety Inventory
